# Prevalence of sleep disorder diagnoses and sleep medication prescriptions in individuals with ADHD across the lifespan: a Swedish nationwide register-based study

**DOI:** 10.1136/bmjment-2023-300809

**Published:** 2023-09-01

**Authors:** Rickard Ahlberg, Miguel Garcia-Argibay, Marc Taylor, Paul Lichtenstein, Brian M D'Onofrio, Agniezska Butwicka, Catherine Hill, Samuele Cortese, Henrik Larsson, Ebba Du Rietz

**Affiliations:** 1 School of Medical Sciences, Örebro University, Orebro, Sweden; 2 Department of Medical Epidemiology and Biostatistics, Karolinska Institute, Stockholm, Sweden; 3 Department of Psychological and Brain Sciences, Indiana University Bloomington, Bloomington, Indiana, USA; 4 Child and Adolescent Psychiatry, Stockholm Health Care Services, Region Stockholm, Stockholm, Sweden; 5 School of Clinical and Experimental Sciences, Faculty of Medicine, University of Southampton, Southampton, UK; 6 Department of Sleep Medicine, Southampton Children's Hospital, University Hospital Southampton NHS Foundation Trust, Southampton, UK, Southampton, UK; 7 Centre for Innovation in Mental Health, School of Psychology, University of Southampton Faculty of Environmental and Life Sciences, Southampton, UK; 8 Solent NHS Trust; Division of Psychiatry and Applied Psychology, School of Medicine, University of Nottingham, Nottingham, UK

**Keywords:** Child & adolescent psychiatry, Adult psychiatry

## Abstract

**Background:**

Consistent evidence suggests a strong association between attention-deficit/hyperactivity disorder (ADHD) and subjectively reported sleep problems. However, the prevalence of clinically ascertained sleep disorder diagnoses and sleep medication prescriptions in individuals with ADHD remains unclear.

**Objective:**

To determine the rates of sleep disorder diagnoses and sleep medication prescriptions in children, adolescents and adults with ADHD.

**Methods:**

We linked Swedish national registers to create a cohort of individuals born 1945–2008. We estimated the absolute and relative risks (using logistic regression models) of different sleep disorder diagnoses and medication prescriptions in individuals with and without ADHD. The analyses were performed across five different age groups: children (5–11 years), adolescents (12–17 years), young adults (18–30 years), middle-aged adults (31–45 years) and older adults (46–60 years).

**Findings:**

Among individuals with ADHD (N=145 490, 2.25% of the cohort), 7.5% had a sleep disorder diagnosis and 47.5% had been prescribed sleep medication. Individuals with ADHD, across all age groups, had a statistically significantly increased risk of having any sleep disorder diagnosis (OR_range_=6.4–16.1) and any sleep medication prescription (OR_range_=12.0–129.4) compared with individuals without ADHD. While rates of sleep disorders were highest in older adults, the relative risks were highest in youth.

**Conclusions:**

Individuals with ADHD have a substantially increased risk of sleep disorder diagnoses and sleep medication prescriptions, from childhood into older adulthood.

**Clinical implications:**

More clinical efforts are needed to tackle impairing sleep problems in individuals with ADHD via systematic sleep assessment, appropriate diagnosis, and pharmacological and non-pharmacological interventions. Sleep medication use should be informed by sleep disorder diagnosis.

**WHAT IS ALREADY KNOWN ON THIS TOPIC**

- Studies mainly in children and adolescents show a significant association between attention-deficit/hyperactivity disorder (ADHD) and subjectively reported (eg, parent report) sleep problems and, to a lesser extent, objective (eg, wrist worn actigraphy) sleep problems.
- However, the prevalence of clinically ascertained sleep disorders and medications for sleep disorders in individuals with ADHD through the lifespan is unknown.

**WHAT THIS STUDY ADDS**

- In this large nationwide register study (N=6 470 658), we found that 7.5% of individuals with ADHD had a formal sleep disorder diagnosis and 47.5% had been prescribed medication(s) for sleep disorders.
- This corresponded to an 8-fold increased risk of having a sleep disorder diagnosis and a substantial 14-fold increased risk of having sleep medication prescribed compared with individuals without ADHD.
- The prevalence of sleep disorders was highest in the middle-aged and older adult cohorts, while the relative risks were highest in the child and adolescent cohorts.
- The prevalence rates of clinically diagnosed sleep disorders in individuals with ADHD were substantially lower than the rates of medication prescriptions for sleep.

**HOW THIS STUDY MIGHT AFFECT RESEARCH, PRACTICE OR POLICY**

- This study provides, for the first time, the prevalence rates of sleep disorder diagnoses and sleep medication use in a national study of individuals with ADHD.
- The findings highlight the need for greater clinical efforts on prevention, diagnosis and adequate treatments for sleep disorders in individuals with ADHD, from childhood to older adulthood.
- The findings also point to the need for sleep medication to be informed by sleep disorder diagnosis.

## Background

Attention-deficit/hyperactivity disorder (ADHD) is a neurodevelopmental condition characterised by impairing levels of inattention and/or hyperactivity/impulsivity. The prevalence of ADHD is around 5% in youth and 2.5% in adults.^1^ ADHD often co-occurs with other mental and physical disorders,^1^ and one of the most frequently reported co-occurring issues is sleep problems.^2^ Adequate sleep is essential for maintaining optimal mental and physical health. Indeed, robust associations have been found between poor quality or restricted sleep and increased risk for cardiometabolic issues, low mood, anxiety and impaired cognitive performance.^3 4^ Furthermore, sleep problems may also exacerbate symptoms of ADHD.^5^

Most of the studies to date on the association between ADHD and sleep problems have used subjective, such as self-rating scales, and objective, including wrist-worn actigraph measures of sleep parameters. Systematic reviews and meta-analyses of studies based on such subjective and objective measures of sleep have reported significant associations between ADHD and sleep alterations, which often present themselves in early childhood.^6–9^ Previous studies are characterised by substantial differences in study populations (eg, children or adults), definitions of ADHD (eg, symptoms or diagnoses) and sleep outcomes (eg, subjective or objective, persistent or transient). While these associations are valuable for understanding the link between ADHD and different aspects of poor sleep, there is a lack of studies providing evidence on the prevalence of clinically diagnosed sleep disorders, as well as of medication prescriptions, in individuals with ADHD. Furthermore, previous studies on sleep problems in ADHD have mainly been conducted among children and adolescents and using relatively small clinical samples with limited generalisability.^6 7^ Thus, much less is known about disordered sleep in adults with ADHD and how the rates of different sleep disorder diagnoses may change over the lifespan. Large population-based cohort studies are therefore needed to examine the prevalence rates of different sleep disorders and medical prescriptions in ADHD across different age groups from childhood to late adulthood.

Determining the prevalence rates of diagnosed sleep problems in individuals with ADHD and gaining a better understanding of the trends in clinical practices across different age groups may lead to more targeted efforts to assess and prevent the development of sleep problems in ADHD and to improve treatment efforts. Greater knowledge of the prevalence of sleep disorders, and the patterns of sleep medication used, in individuals with ADHD is therefore important for public health and healthcare planning.

### Objective

The objective of this study was to help fill the current research gaps by rigorously assessing the prevalence rates of a comprehensive set of sleep disorders, using clinically ascertained diagnoses and medication prescriptions, in children, adolescents and adults with and without ADHD using a large population-based register study. To our knowledge, this is the first study examining the prevalence of clinically assigned sleep disorders in individuals with ADHD in a nationally representative cohort from childhood to older adulthood.

We hypothesised that the risk of sleep disorders would be overall greatly increased in individuals with ADHD, from childhood to older adulthood, and across females and males. As there is limited research directly comparing age and sex differences in the association of sleep disorders with ADHD, and findings have been mixed,^8 10^ it is important to outline whether there are substantial group differences in the risks of sleep disorders. Furthermore, medications for ADHD can have negative effects on sleep quality (such as on insomnia)^6 9^; however, limited available research suggests that the association between ADHD and sleep problems is not solely driven by the effects of medication for ADHD.^6 8^ We therefore hypothesised that even individuals who are not on medications for ADHD will show increased rates of sleep disorder diagnoses and medication prescriptions for sleep.

## Methods

### Study design and study population

We used the Swedish Total Population Register to create a cohort of 6 470 658 individuals born between 1945 and 2008. Individuals had to be alive and living in Sweden during the study period (up to year 2013). The Swedish Total Population register was linked to the Swedish National Patient Register (NPR), which includes inpatient hospitalisations from 1975 to 2013 and outpatient specialist diagnoses from 2001 to 2013.^11^ All individuals with at least one diagnosis of ADHD in the NPR were identified by using International Classification of Diseases-10 (ICD-10: code F90). Individuals with ADHD were also identified by dispensions of ADHD medications (Anatomical Therapeutic Chemical Classification System codes (ATC codes: N06BA01/N06BA02/N06BA04/N06BA09/N06BA12) in the Prescribed Drug Register (PDR, linkage from 2005 to 2013). Previous research has indicated high specificity for this register-based ADHD definition in Sweden.^12^ The PDR records all prescribed drugs in Sweden from 2005 and onwards.^13^ We studied 10 sleep disorders (eg, insomnia, sleep apnoea, hypersomnia), identified through ICD diagnoses, and 5 medications for sleep (zopiclone, zolpidem, melatonin, propiomazine, zaleplon), obtained via ATC codes for prescriptions (see table 1). ADHD and sleep were treated as lifetime diagnoses (presence/absence).

**Table 1:** Iinternational Classification of Diseases-10 (ICD-10) diagnostic codes for sleep disorders and Anatomical Therapeutic Chemical Classification System codes (ATC codes) for medication prescriptions for sleep

ICD 10 diagnoses (inpatient/outpatient)	Codes
Insomnia	F51.0, G47.0
Hypersomnia	F51.1, G47.1
Disorder of sleep-wake schedule (circadian rhythm)	F51.2, G47.2
Sleepwalking	F51.3
Night terrors	F51.4
Nightmares	F51.5
Other specified sleep disorders	F51.8, G47.8
Other sleep disorders, unspecified	F51.9, G47.9
Restless legs syndrome	G25.8
Sleep apnoea	G47.3
Narcolepsy and cataplexy	G47.4
**ATC medication prescriptions**	**Codes**
*Sleep disorder as primary indication*	
Zopiclone	N05CF01
Zolpidem	N05CF02
Melatonin	N05CH01
Propiomazine	N05CM06
Zaleplon	N05CF03

### Statistical analyses

We first estimated the absolute risks (ie, lifetime prevalence rates) of sleep disorders and sleep medication prescriptions in individuals with ADHD compared with those without. We then estimated the relative risks of sleep disorders and sleep medications prescriptions in individuals with and without ADHD using logistic regression models, reporting ORs and 95% CIs. Analyses were performed in the full cohort and then stratified across the five age groups based on age at point of study: children (born 1993–2008, followed up between 5 and 11 years), adolescents (born 1988–2000, followed up between 12 and 17 years), young adults (born 1975–1995, followed up between 18 and 30 years), middle-aged adults (born 1960–1982, followed up between 31 and 45 years) and older adults (born 1945–1973, followed up between 46 and 60 years).

The main analyses across the full cohort were further stratified by sex to study whether the ADHD-sleep associations differed in females and males. In a sensitivity analysis, we re-ran the main analyses by defining the ADHD group as individuals with an ADHD diagnosis who had not received ADHD medication, to investigate whether the ADHD-sleep associations could be driven by ADHD medication use. All statistical models adjusted for birth year and sex, except the models that were stratified by age group or sex, respectively. Data management and statistical analyses were performed using SAS software V.9.4 (SAS Institute) and Stata V.15.

### Findings

#### Descriptive statistics

**Figure 1:**
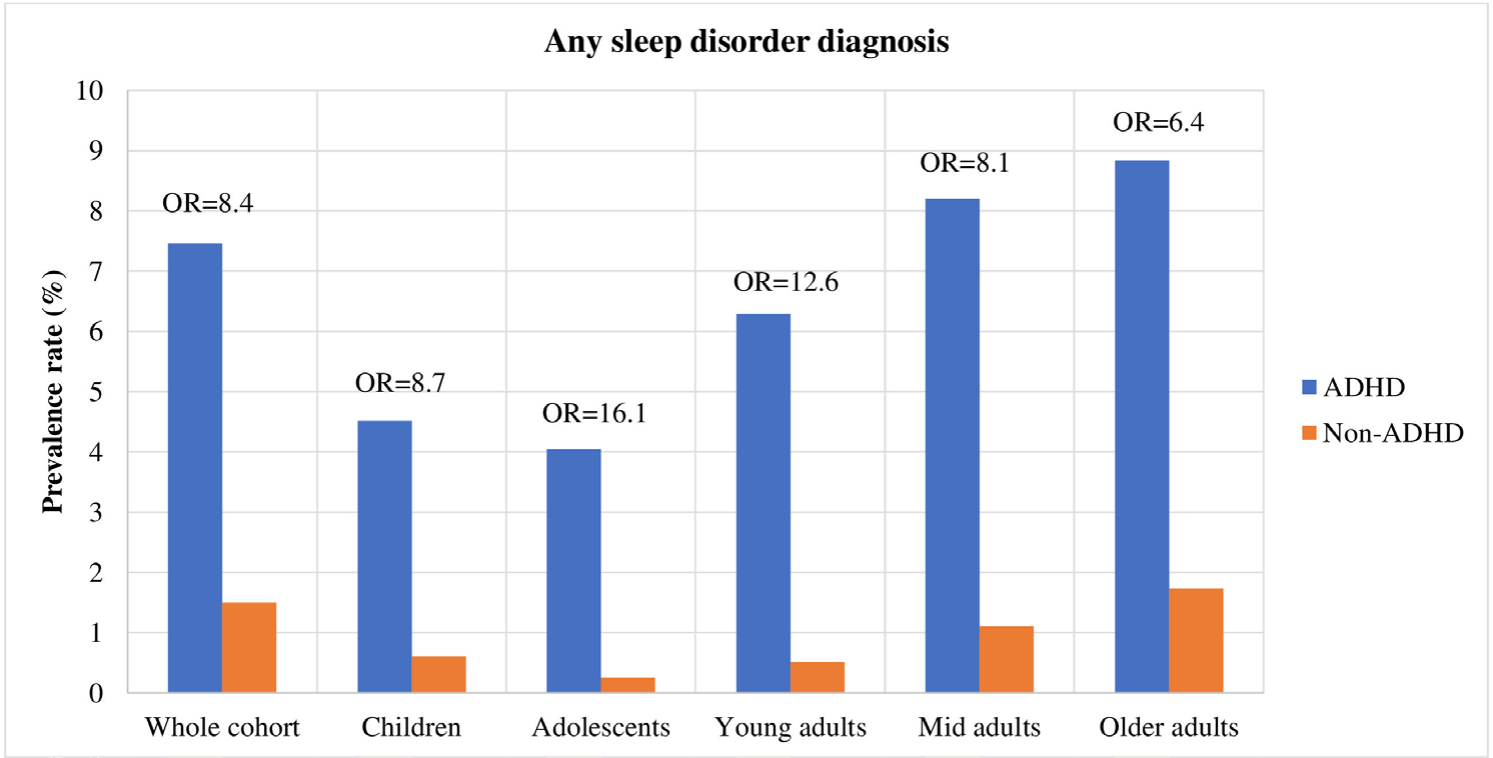
Absolute and relative rates of lifetime sleep disorder diagnoses in individuals with and without attention-deficit/hyperactivity disorder (ADHD). OR=Odds ratio.

**Figure 2:**
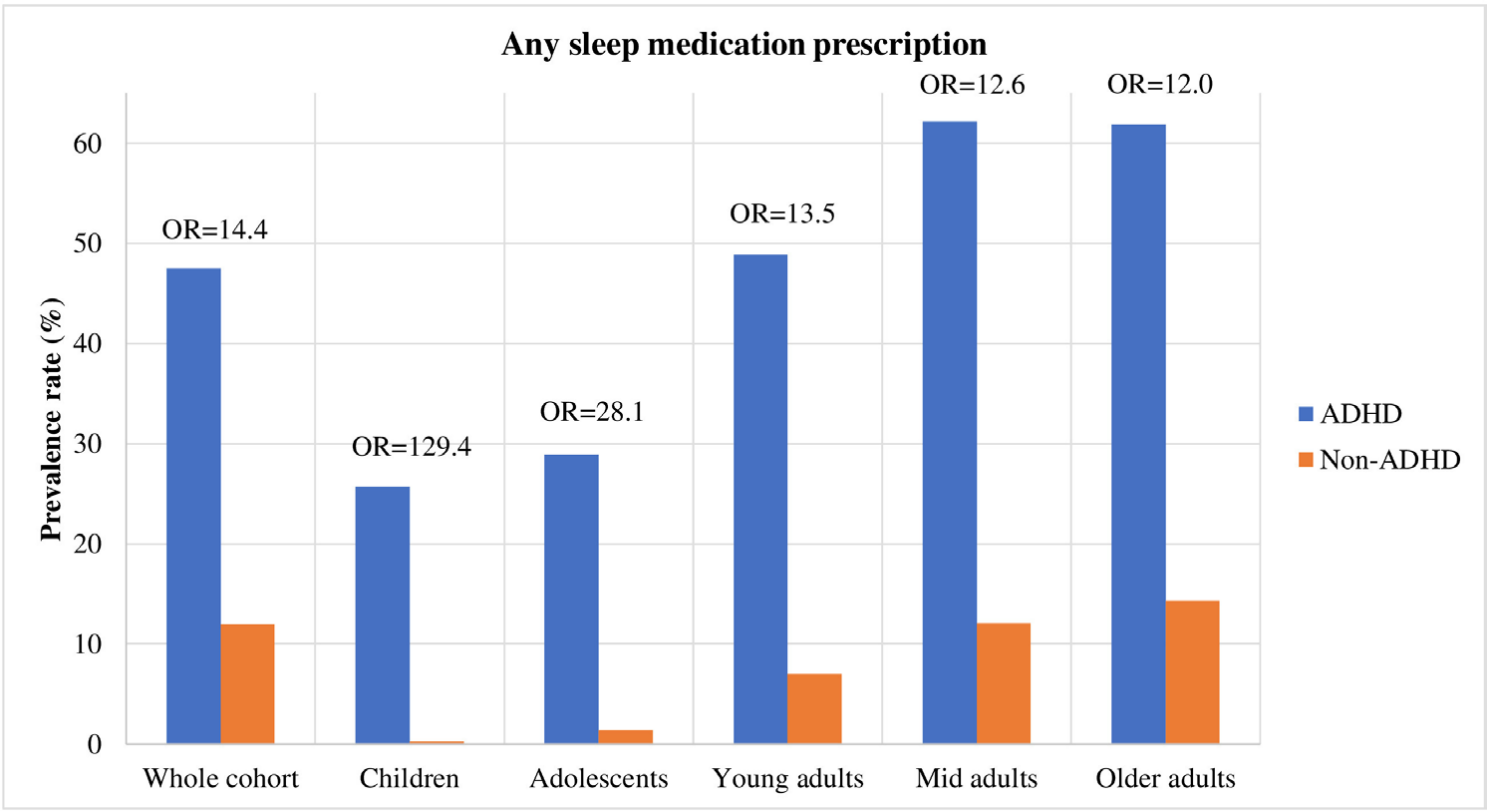
Absolute and relative rates of sleep medication prescriptions in individuals with and without attention-deficit/hyperactivity disorder (ADHD). OR=Odds ratio.

The full cohort consisted of 6 470 658 residents in Sweden followed up between ages 5 and 60 years (born between 1945 and 2008), of which 145 490 had an ADHD diagnosis (2.25%). Among individuals with ADHD, 7.5% had a formal sleep disorder diagnosis and 47.5% had been prescribed sleep medication, compared with 1.5% and 12%, respectively, in individuals without ADHD. The prevalence rates of sleep disorder diagnoses and sleep medication prescriptions were highest in the older adult age cohort, relative to the younger cohorts, in both individuals with and without ADHD (figures 1,2). The most common types of sleep diagnoses in the full cohort were unspecified sleep disorder (prevalence: 3.84% in ADHD group, 0.37% non-ADHD group), insomnia (1.71% in ADHD group, 0.14% non-ADHD group) and sleep apnoea (1.27% in ADHD group, 0.96% non-ADHD group). While unspecified sleep disorders was the most frequently diagnosed sleep condition in the younger ADHD cohorts, sleep apnoea was the most common in the oldest cohort (table 2).

**Table 2:** List of most common types of sleep disorder diagnoses in individuals with and without ADHD across age cohorts

	Most common type of sleep diagnoses		
	1st	2nd	3rd
ADHD group			
Children	Unspecified (2.66%)	Insomnia (1.03%)	Apnoea (0.43%)
Adolescents	Unspecified (2.15%)	Insomnia (1.19%)	Sleep wake schedule (0.50%)
Young adults	Unspecified (3.55%)	Insomnia (1.43%)	Sleep wake schedule (0.54%)
Mid adults	Unspecified (3.51%)	Apnoea (2.51%)	Insomnia (1.45%)
Older adults	Apnoea (3.84%)	Unspecified (2.62%)	Insomnia (1.36%)
Full cohort	Unspecified (3.84%)	Insomnia (1.71%)	Apnoea (1.27%)
Non-ADHD group			
Children	Unspecified (0.32 %)	Apnoea (0.16 %)	Insomnia (0.09 %)
Adolescents	Unspecified (0.14 %)	Insomnia (0.05 %)	Apnoea (0.03 %)
Young adults	Unspecified (0.23 %)	Apnoea (0.15 %)	Insomnia (0.08 %)
Mid adults	Apnoea (0.71 %)	Unspecified (0.25 %)	Insomnia (0.10 %)
Older adults	Apnoea (1.39 %)	Unspecified (0.22 %)	Insomnia (0.10 %)
Full cohort	Apnoea (0.96%)	Unspecified (0.37%)	Insomnia (0.14%)

Note: Prevalence rates of diagnoses displayed in parentheses. Children (age at point of study between 5 and 11 years), adolescents (followed up between 12 and 17 years), young adults (followed up between 18 and 30 years), middle-aged adults (followed up between 31 and 45 years) and older adults (followed up between 46 and 60 years).

ADHD, attention-deficit/hyperactivity disorder.

#### Association of ADHD with sleep disorders and sleep medications

Individuals with ADHD had a statistically significant increased risk of having any sleep disorder diagnosis compared with individuals without ADHD (OR=8.38 (95% CI 8.20 to 8.57)), and the ORs for individual sleep disorder diagnoses ranged from 2.66 to 20.18 (figure 1, table 3). Individuals with ADHD also had a statistically significant increased risk of being prescribed a sleep medication (OR=14.41 (95% CI 14.24 to 14.58)); the ORs of individual medication prescriptions ranged from 6.89 to 37.73 (figure 2, table 3). The highest relative risks for individual sleep disorders in individuals with ADHD were seen for hypersomnia (OR=20.18 (95% CI 18.18 to 22.40)), sleep-wake disorder (OR=19.28 (95% CI 17.70 to 21.00)) and insomnia (OR=16.39 (95% CI 15.63 to 17.17)). The highest relative risk for individual sleep medications were seen for melatonin (OR=37.73 (95% CI 37.15 to 38.31)), zaleplon (OR=10.62 (95% CI 10.14 to 11.11)) and propiomazine (OR=8.28 (95% CI 8.17 to 8.40)) (table 3).

**Table 3:** Absolute and relative rates of lifetime sleep disorder diagnoses and sleep medication prescriptions in individuals with and without ADHD (5–60 years)

Full cohort (born 1945–2008)						
ADHD prevalence=2.25%	ADHD group (n=145 490)		Non-ADHD group (n=632 5168)		OR	95% CI
	**N**	**%**	**N**	**%**		
Diagnosed sleep disorder						
Insomnia	2492	1.71	8644	0.14	16.39	15.63 to 17.17
Hypersomnia	523	0.36	1525	0.02	20.18	18.18 to 22.40
Sleepwalk	70	0.05	436	0.01	6.31	4.88 to 8.17
Sleep-wake schedule (circadian rhythm)	834	0.57	1852	0.03	19.28	17.70 to 21.00
Sleep terror	57	0.04	329	0.01	4.80	3.61 to 6.37
Nightmares	60	0.04	347	0.01	7.31	5.52 to 9.67
Other	303	0.21	1174	0.02	12.71	11.14 to 14.49
Unspecified	5584	3.84	23 289	0.37	12.37	12.00 to 12.76
RLS	276	0.19	3535	0.06	6.85	6.04 to 7.76
Sleep apnoea	1853	1.27	60 772	0.96	2.66	2.54 to 2.80
Narcolepsy and cataplexy	647	0.44	461	0.01	73.12	64.44 to 82.96
*Any sleep disorder*	10 856	*7.46*	95 075	*1.50*	*8.38*	*8.20 to 8.57*
Medication prescription						
Zopiclone	29 935	20.58	403 688	6.38	7.93	7.82 to 8.05
Zolpidem	20 296	13.95	298 378	4.72	6.89	6.78 to 7.01
Melatonin	35 433	24.35	56 969	0.90	37.73	37.15 to 38.31
Propiomazine	31 623	21.74	357 999	5.66	8.28	8.17 to 8.40
Zaleplon	2277	1.57	17 626	0.28	10.62	10.14 to 11.11
*Any sleep medication*	69 081	*47.48*	757 782	*11.98*	*14.41*	*14.24 to 14.58*

ADHD, attention-deficit/hyperactivity disorder; RLS, restless leg syndrome.

#### Association of ADHD with sleep disorders and sleep medications across age groups

There was an increased risk of having any sleep disorder diagnosis in individuals with ADHD across all age groups (OR_range_=6.35–16.13; figure 1 and online supplemental tables 1–5). The relative risk for any sleep disorder in the ADHD group compared with the control group was highest in the adolescent cohort (OR=16.13 (95% CI 15.21 to 17.10)), followed by the young adult cohort (OR=12.59 (95% CI 12.08 to 13.12)).

10.1136/bmjment-2023-300809.supp1 Supplementary data

There was also an increased risk of having any sleep medication prescription in individuals with ADHD across all age groups (OR range=12.03–129.35; figure 2 and online supplemental tables 1–5). However, children with ADHD had the highest relative risk of having sleep medication prescribed (OR=129.35 (95% CI 124.45 to 134.45)), followed by adolescents (OR=28.10 (95% CI 27.39 to 28.83)).

#### Sex-specific associations of ADHD with sleep disorders and sleep medication

In females with ADHD, 8.39% had any sleep disorder diagnosis and 55.80% had been prescribed any sleep medication. In males with ADHD, 6.91% had any sleep disorder diagnosis and 42.58% had been prescribed any sleep medication (online supplemental tables 6,7). Females with ADHD relative to those without ADHD (OR=11.21 (95% CI 10.85 to 11.59)) had a significantly higher risk for any sleep disorder compared with males (OR=7.28 (95% CI 7.08 to 7.49)) (non-overlapping 95% CIs). The relative risk for being prescribed any sleep medication was similar between females and males (females OR=14.03 (95% CI 13.77 to 14.29); males OR=14.17 (95% CI 13.96 to 14.39)).

### Sensitivity analyses

When we re-ran the main analyses excluding individuals who had received an ADHD medication prescription from the ADHD group, we still found a significantly increased risk, although attenuated, in individuals with ADHD of being diagnosed with any sleep disorder (OR=6.71 (95% CI 6.32 to 7.11)) and prescribed any sleep medication (OR=7.11 (95% CI 6.89 to 7.34)) (online supplemental table 8). These effects were significantly smaller than in the main analyses where the ADHD group also consisted of individuals who had been prescribed ADHD medication (non-overlapping 95% CIs).

## Discussion

To our knowledge, this is the first study examining the prevalence of clinically assigned sleep disorders (ICD diagnoses) as well as of sleep medication prescriptions in individuals with and without ADHD in a nationally representative cohort from childhood to older adulthood. In this study based on 6.4 million individuals, of whom 145 490 had an ADHD diagnosis, we found that ADHD was significantly associated with an excess risk of sleep disorders from childhood to older adulthood. Across the whole cohort, 7.5% of individuals with ADHD had any sleep disorder diagnosis, in contrast to 1.5% of individuals without ADHD. This corresponds to an overall eightfold higher risk for sleep disorders in individuals with ADHD. The prevalence of sleep disorders in ADHD was highest in the middle-aged and older adult cohorts, reflecting that absolute risk may increase over time. However, the relative risks were highest in the child and adolescent cohorts, reflecting the very low prevalence of sleep disorder diagnoses and medication prescriptions in young individuals without ADHD. In a clinical context, this suggests that individuals with ADHD should be systematically screened for sleep disorders across the lifespan, from childhood to older adulthood. Our results also point to the importance of a lifespan perspective in research on sleep disorders in ADHD.

### Prevalence of specific sleep disorders in individuals with ADHD

The prevalence rates we reported in this study for individual types of sleep disorders in ADHD are much lower than the rates reported in clinical studies using best estimate clinical evaluations based on a combination of validated self-reports, structured interviews and chart review.^14 15^ Previous clinical studies have, for example, reported that, when systematically assessed, around 22% of children and 44%–67% of adults with ADHD meet the criteria for insomnia disorder, compared with our prevalence rates of 1.03%–1.45%.^14–16^ The markedly lower administrative prevalence of diagnosed sleep disorders in our study could be an indication that sleep disorders are being underdiagnosed in individuals with ADHD, in contrast to the much higher rates of medication prescriptions for sleep problems. Indeed, underdiagnosis of sleep disorders is a common problem in clinical practice in general,^17 18^ possibly because of limited coverage of sleep disorders in clinical psychology programmes and in medical school.^19–21^

The most common sleep disorder diagnosis in individuals with ADHD across all age cohorts, except the older-aged adults, was sleep disorder ‘unspecified’, with prevalence rates ranging from 2.2% to 3.6% (table 2). For older-aged adults with ADHD, the most common sleep disorder diagnosis was sleep apnoea, with a prevalence of 3.8%. The high rates of ‘unspecified’ diagnoses may reflect other sleep disorders (such as RLS or sleep-wake schedule disorders (ie, circadian rhythm disorders)) that can easily be missed by practitioners or misdiagnosed as more general sleep disorders, such as insomnia or unspecified sleep disorders.^20^ Practitioners who are not sleep experts may have inadequate knowledge or tools to assess specific sleep disorders, which is in line with previous reports of poor coverage of sleep disorders in clinical psychology programmes and in medical school education.^19–21^ Misdiagnosis of sleep disorders can be very serious, as treatments to alleviate sleep problems are then ineffective and can potentially be harmful for patients.^22^

### Lifespan associations between ADHD, sleep disorders and sleep medications

The risk of being diagnosed with a sleep disorder and receiving medication prescriptions was elevated across all age groups for individuals with ADHD compared with those without. The prevalence rates of having any sleep disorder diagnosis or any sleep medication prescription were highest in the oldest cohorts. This is in accordance with a previous clinical chart review study in 1828 children, young adults and older adults with ADHD,^23^ where sleep problems were more commonly self-reported in older adults with ADHD compared with children and youth. This pattern is also in line with clinical studies using best estimate diagnoses by psychiatrists and psychologist.^14 15^ However, while the absolute risk of sleep problems was highest in the older cohorts, the relative risk of any sleep disorder was highest in the adolescent cohort in our study (16-fold increased rate in those with ADHD), followed by the child cohort (9-fold increased rate). Children with ADHD had the highest relative risk of having a prescription for sleep medication with a 129-fold higher risk compared with peers without ADHD (25.7% of children with and 0.26% of children without ADHD). This high estimate is likely reflecting the fact that sleep medications other than melatonin are seldom prescribed to children. Similar estimates were found in a recent Danish population-based study, where 29.2% of children with ADHD had sleep problems as defined through prescribed melatonin and/or a registered sleep disorder diagnosis.^24^ The most commonly prescribed sleep medication in children and adolescents in our study was melatonin (available via prescription only in Sweden during the study period), while propiomazine and zopiclone were more commonly prescribed in the older cohorts.

### Sex-specific associations between sleep disorders, sleep medication and ADHD

Females with ADHD had both higher absolute and relative risk of any sleep disorder diagnosis compared with males with ADHD. This is in accordance with earlier clinical studies finding higher rates of sleep problems in girls and women compared with boys and men,^23 25^ although findings from the literature have been mixed and overall limited.^8 23 25^ Previous literature has often reported that females with ADHD have a higher relative risk of comorbidities compared with males^8^; however, the reason for this sex difference is largely unknown. Females also had higher rates of sleep medication prescriptions than males, but the relative risk of being prescribed sleep medication was similar across sexes. This is in accordance with other studies which have found a higher rate of sleep medications in girls and young women with ADHD but also a more general pattern with higher use of medications of sedatives than men.^26 27^

### Potential impact of ADHD medication on the prevalence of sleep disorders in individuals with ADHD

In the sensitivity analyses where we excluded individuals who had received ADHD medication from the ADHD group, we found that the ADHD-sleep associations attenuated, even though they still remained significant. In particular, there was a substantial attenuation of the relative risk of being prescribed any sleep medication in the ADHD group (OR=7.11 vs 14.41), mainly driven by an attenuation of melatonin prescriptions. There are several possible reasons for this attenuation of effect. First, individuals treated with ADHD medication have more severe ADHD symptoms, which in turn leads to more sleep problems or may reflect a higher comorbidity with sleep problems. Second, individuals treated with ADHD medication are more likely to receive other medications, such as sleep medications. Third, ADHD medications, particularly stimulants, have a negative impact on sleep, as shown by meta-analytic evidence.^28^ Further investigation is required to uncover why stronger ADHD-sleep associations are found in individuals with ADHD who have also received ADHD medications. It is, however, important to note that there was still a significant association between ADHD and sleep disorders after excluding individuals on ADHD medication, which shows that the association is not solely driven by side effects of medication.

### Strengths and limitations

This is the first large-scale study using a representative population cohort to examine the prevalence different types of sleep disorders in ADHD across the lifespan. It is important, however, to interpret results in the context of the limitations of the study. First, we relied on register-based diagnoses of ADHD and sleep disorders, and therefore findings refer to diagnosed patients who are potentially more severely affected than individuals who do not receive or seek healthcare support. Further, the prevalence estimates of sleep disorders were likely underestimated as the medication prescription rates were much higher than the diagnostic prevalence rates. Relatively low rates of clinically ascertained sleep disorder diagnoses are common in registry-based studies.^24 27^ In a registry study on melatonin use in Stockholm in 2016, 1.4% of children aged 6–12 years were prescribed melatonin; however, only 20% of those who prescribed melatonin had a recorded ICD diagnosis of sleep disorder.^27^ We saw the same pattern in our study, where 25.5% of children 5–11 years in the ADHD group had a prescription of melatonin but only 4.5% had a recorded sleep disorder diagnosis. One reason for the relatively low prevalence rates of sleep disorder diagnoses in our study could be that sleep disorders are often treated (and diagnosed) in primary care, which we did not have data. The previous study on melatonin use, however, had data from both psychiatric and primary care and still reported substantially lower rates of diagnosed sleep disorders than sleep medication prescriptions.^27^ The rates of medication prescriptions for sleep may also have been underestimated in our study, due to the common use of off-label medications, such as antihistamines and antidepressants, to treat sleep problems.^29^ We also did not have available information in the registers on non-pharmacological treatment of sleep disorders.

During the follow-up period, there were changes in diagnostic practice for ADHD (eg, rate of ADHD diagnoses increased fivefold between 2004 and 2015),^30^ and the coverage of the patient register has improved over time. Incomplete coverage by the register might introduce outcome misclassification bias (ie, false negatives), which would be most likely to bias estimates towards the null. Thus, our findings might reflect conservative estimates of true associations between ADHD and sleep disorders.

### Clinical implications

We found a much higher prevalence of any sleep medication prescription (47.5%) than sleep disorder diagnoses (7.5%) in individuals with ADHD. This could indicate that clinicians are prescribing sleep medications based on the presence of sleep problems rather than on clinically impairing sleep disorders or formally diagnosed disorders, or that a sizeable portion of individuals with ADHD had sleep symptoms that were impairing but did not meet the criteria for a disorder. However, the prevalence of sleep disorders reported in this study is much lower than in clinical studies using best estimate sleep disorder diagnoses made by psychiatrist/psychologists using self-reports, structured interviews and clinical evaluations in individuals with ADHD (eg, 1.03%–1.45% vs 22%–67% for insomnia).^14–16^ This large discrepancy could be an indication that clinicians, on average, are not conducting evidence-based assessments of sleep disorders in individuals with ADHD. This could reflect a more general pattern where sleep disorders are often underinvestigated or completely ignored,^17 18^ which may be driven by the relatively poor coverage of sleep in medical school education and in clinical psychology programmes.^19–21^ It is possible that sleep problems are seen as symptoms of ADHD or related problems, or are being overshadowed by ADHD, and therefore do not result in a separate sleep diagnosis. Our findings highlight the need for sleep problems to be rigorously assessed and appropriately diagnosed in both men and women with ADHD, throughout the lifespan. Our findings also suggest that greater clinical attention should be directed towards addressing sleep problems in individuals with ADHD. This entails implementing proactive measures through sleep education programmes and providing both pharmacological and non-pharmacological approaches such as cognitive behavioural therapy and parental sleep training.

## Data Availability

Data may be obtained from a third party and are not publicly available. Data cannot be shared publicly due to the Swedish Secrecy Act. Data from Swedish national registers were used for this study and made available by ethical approval. Researchers may apply for access through the Swedish Research Ethics Boards (www.etikprovningsmyndigheten.se) and from the primary data owners Statistics Sweden (www.scb.se), and the National Board of Health and Welfare (socialstyrelsen.se), in accordance with Swedish law.
