# Supplementary material for: Prevalence of sleep disorder diagnoses and sleep medication prescriptions in individuals with ADHD across the lifespan: a Swedish nationwide register-based study
Source: BMJ Ment Health. 2023 Sep 1;26(1):e300809. doi: 10.1136/bmjment-2023-300809 (PMC10577710; doi:10.1136/bmjment-2023-300809)
Supplement: Supplementary data [file bmjment-2023-300809supp001.pdf]

# Prevalence of sleep disorder diagnoses and sleep medication prescriptions in individuals with ADHD across the lifespan: a Swedish nationwide register-based study

## *Supplements*

**Supplementary Table S1:** Absolute and relative rates of sleep disorders and sleep medications in individuals 5-11 years (page 2)

**Supplementary Table S2:** Absolute and relative rates of sleep disorders and sleep medications in individuals 12-17 years (page 3)

**Supplementary Table S3:** Absolute and relative rates of sleep disorders and sleep medications in individuals 18-30 years (page 4)

**Supplementary Table S4:** Absolute and relative rates of sleep disorders and sleep medications in individuals 31-45 years (page 5)

**Supplementary Table S5:** Absolute and relative rates of sleep disorders and sleep medications in individuals 46-60 years (page 6)

**Supplementary Table S6:** Absolute and relative rates of sleep disorders and sleep medications in males with and without ADHD (5-60 years) (page 7)

**Supplementary Table S7:** Absolute and relative rates of sleep disorders and sleep medications in females with and without ADHD (5-60 years) (page 8)

**Supplementary Table S8:** Absolute and relative rates of sleep disorders and sleep medications in individuals with and without ADHD (5-60 years): excluding individuals in the ADHD group who have been prescribed ADHD medication (page 9)



Ahlberg R, *et al. BMJ Ment Health* 2024; 26:e300809. doi: 10.1136/bmjment-2023-300809

**Table S1:** Absolute and relative rates of sleep disorders and sleep medications in individuals 5-11 years.

| **CHILD COHORT (Born 1993-2008)** | | | | | | | |
|---|---|---|---|---|---|---|---|
| ADHD prev=2.36% | **ADHD group (N=40,474)** | | **Non-ADHD group (N=1671013)** | | **OR** | **95% Lower** | **95% Upper** |
| **Diagnosed sleep disorder** | **N** | **%** | **N** | **%** | | | |
| Insomnia | 416 | 1.03 | 1581 | 0.09 | 16.07 | 14.33 | 18.01 |
| Hypersomnia | 24 | 0.06 | 182 | 0.01 | 7.67 | 4.95 | 11.90 |
| Sleepwalk | 17 | 0.04 | 123 | 0.01 | 5.44 | 3.25 | 9.10 |
| Sleep-wake schedule (circadian rhythm) | 128 | 0.32 | 581 | 0.03 | 13.19 | 10.79 | 16.12 |
| Sleep terror | 35 | 0.09 | 251 | 0.02 | 4.92 | 3.44 | 7.03 |
| Nightmares | 9 | 0.02 | 111 | 0.01 | 3.98 | 2.00 | 7.95 |
| Other | 53 | 0.13 | 278 | 0.02 | 10.58 | 7.79 | 14.35 |
| Unspecified | 1076 | 2.66 | 5238 | 0.32 | 10.21 | 9.53 | 10.94 |
| RLS | 16 | 0.04 | 109 | 0.01 | 8.80 | 5.12 | 15.13 |
| Sleep apnoea | 174 | 0.43 | 2615 | 0.16 | 2.59 | 2.22 | 3.03 |
| Narcolepsy and cataplexy | 106 | 0.26 | 243 | 0.01 | 24.66 | 19.36 | 31.42 |
| *Any sleep disorder* | *1829* | *4.52* | *10241* | *0.61* | *8.73* | *8.28* | *9.19* |
| **Medication prescription** | | | | | | | |
| Zopiclone | 35 | 0.09 | 58 | 0.003 | 28.82 | 18.51 | 44.86 |
| Zolpidem | 34 | 0.08 | 52 | 0.003 | 33.27 | 21.03 | 52.64 |
| Melatonin | 10327 | 25.52 | 4202 | 0.25 | 135.03 | 129.83 | 140.44 |
| Propiomazin | 277 | 0.68 | 214 | 0.01 | 56.05 | 46.48 | 67.58 |
| Zaleplon | 1 | 0.002 | 1 | 0 | 68.89 | 3.41 | 1392.42 |
| *Any sleep medication* | *10400* | *25.70* | *4407* | *0.26* | *129.35* | *124.45* | *134.45* |

Note: OR=Odds ratio, 95% CI=95% Confidence interval, RLS=Restless Leg Syndrome.



Ahlberg R, *et al. BMJ Ment Health* 2024; 26:e300809. doi: 10.1136/bmjment-2023-300809

**Table S2:** Absolute and relative rates of sleep disorders and sleep medications in individuals 12-17 years.

| **ADOLESCENT COHORT (Born 1988-2000)** | | | | | | | |
|---|---|---|---|---|---|---|---|
| ADHD prev=3.12% | **ADHD group (N=47017)** | | **Non-ADHD group (N=1457622)** | | **OR** | **95% Lower** | **95% Upper** |
| **Diagnosed sleep disorder** | **N** | **%** | **N** | **%** | | | |
| Insomnia | 558 | 1.19 | 700 | 0.05 | 25.89 | 23.01 | 29.12 |
| Hypersomnia | 38 | 0.08 | 57 | 0.004 | 20.82 | 13.50 | 32.11 |
| Sleepwalk | 7 | 0.01 | 37 | 0.003 | 4.53 | 1.99 | 10.33 |
| Sleep-wake schedule (circadian rhythm) | 234 | 0.50 | 302 | 0.02 | 26.76 | 22.30 | 32.11 |
| Sleep terror | 7 | 0.01 | 23 | 0.002 | 7.42 | 3.10 | 17.74 |
| Nightmares | 8 | 0.02 | 57 | 0.004 | 4.22 | 1.98 | 8.97 |
| Other | 45 | 0.10 | 109 | 0.007 | 11.06 | 7.72 | 15.84 |
| Unspecified | 1011 | 2.15 | 2106 | 0.14 | 14.08 | 13.01 | 15.23 |
| RLS | 9 | 0.02 | 46 | 0.003 | 6.31 | 3.03 | 13.15 |
| Sleep apnoea | 55 | 0.12 | 439 | 0.03 | 3.39 | 2.55 | 4.51 |
| Narcolepsy and cataplexy | 143 | 0.30 | 45 | 0.003 | 96.54 | 68.23 | 136.58 |
| *Any sleep disorder* | *1900* | *4.04* | *3627* | *0.25* | *16.13* | *15.21* | *17.10* |
| **Medication prescription** | | | | | | | |
| Zopiclone | 836 | 1.78 | 3221 | 0.22 | 10.16 | 9.38 | 11.00 |
| Zolpidem | 503 | 1.07 | 2060 | 0.14 | 10.59 | 9.55 | 11.73 |
| Melatonin | 11944 | 25.40 | 10029 | 0.69 | 42.61 | 41.35 | 43.90 |
| Propiomazin | 3031 | 6.44 | 9327 | 0.64 | 12.22 | 11.70 | 12.78 |
| Zaleplon | 14 | 0.03 | 107 | 0.007 | 7.19 | 4.03 | 12.83 |
| *Any sleep medication* | *13600* | *28.93* | *20240* | *1.39* | *28.10* | *27.39* | *28.83* |

Note: OR=Odds ratio, 95% CI=95% Confidence interval, RLS=Restless Leg Syndrome.

**Table S3:** Absolute and relative rates of sleep disorders and sleep medications in individuals 18-30 years.

| **YOUNG ADULT COHORT (1975-1995)** | | | | | | | |
|---|---|---|---|---|---|---|---|
| ADHD prev=1.97% | **ADHD group (N=49516)** | | **Non-ADHD group (N=2462979)** | | **OR** | **95% Lower** | **95% Upper** |
| **Diagnosed sleep disorder** | **N** | **%** | **N** | **%** | | | |
| Insomnia | 707 | 1.43 | 2042 | 0.08 | 17.45 | 15.95 | 19.08 |
| Hypersomnia | 186 | 0.38 | 518 | 0.02 | 18.63 | 15.64 | 22.20 |
| Sleepwalk | 19 | 0.04 | 154 | 0.006 | 6.11 | 3.75 | 9.94 |
| Sleep-wake schedule (circadian rhythm) | 266 | 0.54 | 649 | 0.03 | 17.42 | 15.03 | 20.20 |
| Sleep terror | 5 | 0.01 | 40 | 0.002 | 7.26 | 2.79 | 18.88 |
| Nightmares | 19 | 0.04 | 107 | 0.004 | 9.03 | 5.47 | 14.90 |
| Other | 108 | 0.22 | 332 | 0.01 | 13.53 | 10.83 | 16.92 |
| Unspecified | 1758 | 3.55 | 5662 | 0.23 | 14.55 | 13.76 | 15.39 |
| RLS | 61 | 0.12 | 379 | 0.02 | 8.91 | 6.74 | 11.77 |
| Sleep apnoea | 242 | 0.49 | 3673 | 0.15 | 3.49 | 3.06 | 3.98 |
| Narcolepsy and cataplexy | 215 | 0.43 | 204 | 0.008 | 48.23 | 39.44 | 58.99 |
| *Any sleep disorder* | *3113* | *6.29* | *12517* | *0.51* | *12.59* | *12.08* | *13.12* |
| **Medication prescription** | | | | | | | |
| Zopiclone | 12834 | 25.92 | 82132 | 3.33 | 10.87 | 10.63 | 11.11 |
| Zolpidem | 8515 | 17.19 | 58249 | 2.36 | 10.29 | 10.03 | 10.56 |
| Melatonin | 8933 | 18.04 | 13072 | 0.53 | 32.10 | 31.17 | 33.06 |
| Propiomazin | 13594 | 27.45 | 88088 | 3.58 | 10.44 | 10.22 | 10.67 |
| Zaleplon | 886 | 1.79 | 3897 | 0.16 | 16.58 | 15.34 | 17.92 |
| *Any sleep medication* | *24199* | *48.87* | *172515* | *7.00* | *13.45* | *13.20* | *13.70* |

Note: OR=Odds ratio, 95% CI=95% Confidence interval, RLS=Restless Leg Syndrome.



Ahlberg R, *et al. BMJ Ment Health* 2024; 26:e300809. doi: 10.1136/bmjment-2023-300809

**Table S4:** Absolute and relative rates of sleep disorders and sleep medications in individuals 31-45 years.

| **MIDDLE-AGED ADULT COHORT (1960-1982)** | | | | | | | |
|---|---|---|---|---|---|---|---|
| Prev adhd=0.98% | **ADHD group (N=26865)** | | **Non-ADHD group (N=2716319)** | | **OR** | **95% Lower** | **95% Upper** |
| **Diagnosed sleep disorder** | **N** | **%** | **N** | **%** | | | |
| Insomnia | 390 | 1.45 | 2769 | 0.10 | 14.37 | 12.90 | 16.01 |
| Hypersomnia | 168 | 0.63 | 562 | 0.02 | 29.95 | 25.11 | 35.71 |
| Sleepwalk | 11 | 0.04 | 121 | 0.004 | 8.18 | 4.40 | 15.22 |
| Sleep-wake schedule (circadian rhythm) | 77 | 0.29 | 454 | 0.02 | 16.13 | 12.63 | 20.60 |
| Sleep terror | 8 | 0.03 | 21 | 0 | 32.57 | 14.27 | 74.33 |
| Nightmares | 11 | 0.04 | 71 | 0.002 | 15.29 | 8.04 | 29.09 |
| Other | 46 | 0.17 | 342 | 0.01 | 13.16 | 9.64 | 17.97 |
| Unspecified | 943 | 3.51 | 6806 | 0.25 | 13.57 | 12.65 | 14.55 |
| RLS | 95 | 0.35 | 1188 | 0.04 | 8.44 | 6.83 | 10.42 |
| Sleep apnoea | 675 | 2.51 | 19534 | 0.71 | 3.71 | 3.43 | 4.01 |
| Narcolepsy and cataplexy | 174 | 0.65 | 155 | 0.006 | 117.06 | 93.54 | 146.49 |
| *Any sleep disorder* | *2203* | *8.20* | *29841* | *1.10* | *8.14* | *7.78* | *8.52* |
| **Medication prescription** | | | | | | | |
| Zopiclone | 10837 | 40.34 | 171177 | 6.30 | 10.27 | 10.01 | 10.53 |
| Zolpidem | 7443 | 27.71 | 132840 | 4.89 | 8.01 | 7.79 | 8.24 |
| Melatonin | 4113 | 15.31 | 13772 | 0.51 | 32.12 | 30.93 | 33.36 |
| Propiomazin | 9185 | 34.19 | 147597 | 5.43 | 9.20 | 8.96 | 9.44 |
| Zaleplon | 815 | 3.03 | 7892 | 0.29 | 12.32 | 11.43 | 13.26 |
| *Any sleep medication* | *16695* | *62.14* | *327856* | *12.07* | *12.57* | *12.26* | *12.89* |

Note: OR=Odds ratio, 95% CI=95% Confidence interval, RLS=Restless Leg Syndrome.



Ahlberg R, *et al. BMJ Ment Health* 2024; 26:e300809. doi: 10.1136/bmjment-2023-300809

**Table S5:** Absolute and relative rates of sleep disorders and sleep medications in individuals 46-60 years.

| **OLDER ADULT COHORT (1945-1973)** | | | | | | | |
|---|---|---|---|---|---|---|---|
| Prev adhd=0.40% | **ADHD group (N=13711)** | | **Non-ADHD group (N=3383005)** | | **OR** | **95% Lower** | **95% Upper** |
| **Diagnosed sleep disorder** | **N** | **%** | **N** | **%** | | | |
| Insomnia | 187 | 1.36 | 3247 | 0.10 | 16.28 | 14.03 | 18.91 |
| Hypersomnia | 95 | 0.69 | 431 | 0.01 | 63.34 | 50.47 | 79.50 |
| Sleepwalk | 5 | 0.04 | 52 | 0.002 | 24.93 | 9.91 | 62.69 |
| Sleep-wake schedule (circadian rhythm) | 25 | 0.18 | 374 | 0.01 | 18.42 | 12.26 | 27.69 |
| Sleep terror | 0 | 0 | 15 | 0 | - | | |
| Nightmares | 3 | 0.02 | 70 | 0.002 | 11.84 | 3.71 | 37.81 |
| Other | 24 | 0.18 | 358 | 0.01 | 18.33 | 12.09 | 27.79 |
| Unspecified | 359 | 2.62 | 7385 | 0.22 | 13.19 | 11.85 | 14.69 |
| RLS | 65 | 0.47 | 2430 | 0.07 | 7.65 | 5.97 | 9.80 |
| Sleep apnoea | 527 | 3.84 | 47188 | 1.39 | 3.27 | 2.99 | 3.57 |
| Narcolepsy and cataplexy | 126 | 0.92 | 128 | 0.004 | 356.80 | 273.13 | 466.10 |
| *Any sleep disorder* | *1212* | *8.84* | *58527* | *1.73* | *6.35* | *5.98* | *6.74* |
| **Medication prescription** | | | | | | | |
| Zopiclone | 5560 | 40.55 | 259305 | 7.66 | 9.62 | 9.29 | 9.96 |
| Zolpidem | 3615 | 26.37 | 206663 | 6.11 | 6.81 | 6.55 | 7.08 |
| Melatonin | 1875 | 13.68 | 23496 | 0.69 | 24.82 | 23.58 | 26.11 |
| Propiomazin | 4277 | 31.19 | 195074 | 5.77 | 8.45 | 8.14 | 8.76 |
| Zaleplon | 336 | 2.45 | 10854 | 0.32 | 10.14 | 9.08 | 11.33 |
| *Any sleep medication* | *8485* | *61.88* | *484446* | *14.32* | *12.03* | *11.62* | *12.47* |

Note: OR=Odds ratio, 95% CI=95% Confidence interval, RLS=Restless Leg Syndrome.



Ahlberg R, *et al. BMJ Ment Health* 2024; 26:e300809. doi: 10.1136/bmjment-2023-300809

**Table S6:** Absolute and relative rates of sleep disorders and sleep medications in males with and without ADHD (5-60 years).

| **Males (1945-2008)** | | | | | | | |
|---|---|---|---|---|---|---|---|
| ADHD prev=2.77 % | **ADHD group (N=91,518)** | | **Non-ADHD group (N=3209945)** | | **OR** | **95% Lower** | **95% Upper** |
| **Diagnosed sleep disorder** | **N** | **%** | **N** | **%** | | | |
| Insomnia | 1381 | 1.51 | 3714 | 0.12 | 17.01 | 15.92 | 18.17 |
| Hypersomnia | 234 | 0.26 | 641 | 0.02 | 18.11 | 15.44 | 21.25 |
| Sleepwalk | 42 | 0.05 | 253 | 0.008 | 5.07 | 3.63 | 7.07 |
| Sleep-wake schedule (circadian rhythm) | 540 | 0.59 | 981 | 0.03 | 18.66 | 16.71 | 20.84 |
| Sleep terror | 41 | 0.05 | 217 | 0.007 | 4.28 | 3.06 | 5.98 |
| Nightmares | 29 | 0.03 | 166 | 0.005 | 6.12 | 4.08 | 9.18 |
| Other | 167 | 0.18 | 614 | 0.02 | 11.03 | 9.22 | 13.20 |
| Unspecified | 3166 | 3.46 | 11332 | 0.35 | 11.85 | 11.36 | 12.36 |
| RLS | 127 | 0.14 | 1407 | 0.04 | 6.78 | 5.61 | 8.17 |
| Sleep apnoea | 1332 | 1.46 | 45815 | 1.43 | 2.40 | 2.27 | 2.54 |
| Narcolepsy and cataplexy | *294* | *0.32* | *201* | *0.01* | *59.70* | *49.22* | *72.42* |
| *Any sleep disorder* | *6328* | *6.91* | *61120* | *1.90* | *7.28* | *7.08* | *7.49* |
| **Medication prescription** | | | | | | | |
| Zopiclone | 14721 | 16.09 | 158108 | 4.70 | 7.63 | 7.48 | 7.78 |
| Zolpidem | 9723 | 10.62 | 113881 | 3.55 | 6.66 | 6.51 | 6.82 |
| Melatonin | 21742 | 23.76 | 22870 | 0.71 | 40.54 | 39.70 | 41.40 |
| Propiomazin | 15715 | 17.17 | 145976 | 4.55 | 7.88 | 7.73 | 8.03 |
| Zaleplon | 1142 | 1.25 | 6817 | 0.21 | 11.31 | 10.58 | 12.09 |
| *Any sleep medication* | *38970* | *42.58* | *305857* | *9.53* | *14.17* | *13.96* | *14.39* |

Note: OR=Odds ratio, 95% CI=95% Confidence interval, RLS=Restless Leg Syndrome.



Ahlberg R, *et al. BMJ Ment Health* 2024; 26:e300809. doi: 10.1136/bmjment-2023-300809

**Table S7:** Absolute and relative rates of sleep disorders and sleep medications in females with and without ADHD (5-60 years).

| **Females (1945-2008)** | | | | | | | |
|---|---|---|---|---|---|---|---|
| ADHD prev=1.70 % | **ADHD group (N=53,972)** | | **Non-ADHD group (N=3115223)** | | **OR** | **95% Lower** | **95% Upper** |
| **Diagnosed sleep disorder** | **N** | **%** | **N** | **%** | | | |
| Insomnia | 1111 | 2.06 | 4930 | 0.16 | 15.75 | 14.72 | 16.84 |
| Hypersomnia | 289 | 0.54 | 884 | 0.03 | 22.45 | 19.57 | 25.74 |
| Sleepwalk | 28 | 0.05 | 183 | 0.006 | 8.83 | 5.91 | 13.20 |
| Sleep-wake schedule (circadian rhythm) | 294 | 0.54 | 871 | 0.03 | 19.89 | 17.37 | 22.76 |
| Sleep terror | 16 | 0.03 | 112 | 0.004 | 6.61 | 3.91 | 11.16 |
| Nightmares | 31 | 0.06 | 181 | 0.006 | 9.18 | 6.26 | 13.49 |
| Other | 136 | 0.25 | 560 | 0.02 | 15.32 | 12.65 | 18.55 |
| Unspecified | 2418 | 4.48 | 11957 | 0.38 | 13.21 | 12.63 | 13.83 |
| RLS | 149 | 0.28 | 2128 | 0.07 | 6.92 | 5.84 | 8.19 |
| Sleep apnoea | 521 | 0.97 | 14957 | 0.48 | 3.68 | 3.37 | 4.02 |
| Narcolepsy and cataplexy | *353* | *0.65* | *260* | *0.01* | *85.30* | *72.25* | *100.69* |
| *Any sleep disorder* | *4528* | *8.39* | *33955* | *1.09* | *11.21* | *10.85* | *11.59* |
| **Medication prescription** | | | | | | | |
| Zopiclone | 15214 | 28.19 | 245580 | 7.89 | 8.25 | 8.09 | 8.43 |
| Zolpidem | 10573 | 19.59 | 184497 | 5.92 | 7.06 | 6.89 | 7.22 |
| Melatonin | 13691 | 25.37 | 33826 | 1.06 | 33.12 | 32.38 | 33.89 |
| Propiomazin | 15908 | 29.47 | 212023 | 6.80 | 8.87 | 8.69 | 9.05 |
| Zaleplon | 1136 | 2.10 | 10,809 | 0.35 | 9.97 | 9.36 | 10.62 |
| *Any sleep medication* | *30111* | *55.80* | *451925* | *14.51* | *14.03* | *13.77* | *14.29* |

Note: OR=Odds ratio, 95% CI=95% Confidence interval, RLS=Restless Leg Syndrome.



Ahlberg R, *et al. BMJ Ment Health* 2024; 26:e300809. doi: 10.1136/bmjment-2023-300809

**Table S8:** Absolute and relative rates of sleep disorders and sleep medications in individuals with and without ADHD (5-60 years): excluding individuals in the ADHD group who have been prescribed ADHD medication

| **WHOLE COHORT (1945-2008)** | | | | | | | |
|---|---|---|---|---|---|---|---|
| ADHD prev=0.29% | **ADHD group (N=19,053)** | | **Non-ADHD group (N=6,325,168)** | | **OR** | **95% Lower** | **95% Upper** |
| **Diagnosed sleep disorder** | **N** | **%** | **N** | **%** | | | |
| Insomnia | 278 | 1.46 | 8644 | 0.14 | 13.81 | 12.24 | 15.59 |
| Hypersomnia | 39 | 0.20 | 1525 | 0.02 | 10.47 | 7.61 | 14.42 |
| Sleepwalk | 9 | 0.05 | 436 | 0.01 | 6.17 | 3.18 | 11.95 |
| Sleep-wake schedule (circadian rhythm) | 91 | 0.48 | 1852 | 0.03 | 16.07 | 13.00 | 19.87 |
| Sleep terror | 8 | 0.04 | 329 | 0.01 | 5.94 | 2.94 | 11.98 |
| Nightmares | 8 | 0.04 | 347 | 0.01 | 7.36 | 3.64 | 14.86 |
| Other | 36 | 0.19 | 1174 | 0.02 | 11.17 | 8.00 | 15.60 |
| Unspecified | 665 | 3.49 | 23289 | 0.37 | 11.00 | 10.16 | 11.90 |
| RLS | 30 | 0.16 | 3535 | 0.06 | 5.30 | 3.70 | 7.61 |
| Sleep apnoea | 243 | 1.28 | 60772 | 0.96 | 2.34 | 2.06 | 2.66 |
| Narcolepsy and cataplexy | | | 461 | 0.01 | 7.75 | 4.14 | 14.54 |
| *Any sleep disorder* | *1242* | *6.52* | *95075* | *1.50* | *6.71* | *6.32* | *7.11* |
| **Medication prescription** | | | | | | | |
| Zopiclone | 3452 | 18.12 | 403688 | 6.38 | 6.08 | 5.84 | 6.32 |
| Zolpidem | 2141 | 11.24 | 298378 | 4.72 | 4.85 | 4.62 | 5.08 |
| Melatonin | 1669 | 8.76 | 56969 | 0.90 | 12.25 | 11.64 | 12.89 |
| Propiomazin | 3741 | 19.63 | 357999 | 5.66 | 6.70 | 6.45 | 6.95 |
| Zaleplon | 207 | 1.09 | 17626 | 0.28 | 6.83 | 5.94 | 7.84 |
| *Any sleep medication* | *6463* | *33.92* | *757782* | *11.98* | *7.11* | *6.89* | *7.34* |

Note: OR=Odds ratio, 95% CI=95% Confidence interval, RLS=Restless Leg Syndrome.
